# Establishment of a novel human T-cell leukemia virus type 1 infection model using cell-free virus

**DOI:** 10.1128/jvi.01862-23

**Published:** 2024-01-31

**Authors:** Koh Nagata, Kenta Tezuka, Madoka Kuramitsu, Naoki Fuchi, Yuri Hasegawa, Isao Hamaguchi, Kiyonori Miura

**Affiliations:** 1 Department of Obstetrics and Gynecology, Nagasaki University Graduate School of Biomedical Sciences, Nagasaki, Japan; 2 Research Center for Biological Products in the Next Generation, National Institute of Infectious Diseases, Tokyo, Japan; Icahn School of Medicine at Mount Sinai, New York, New York, USA

**Keywords:** cell-free HTLV-1 infection, HTLV-1 infectious molecular clone, HTLV-1-producing cells, viral replication cycle, humanized mouse model

## Abstract

The primary mode of infection by human T-cell leukemia virus type 1 (HTLV-1) is cell-to-cell transmission during contact between infected cells and target cells. Cell-free HTLV-1 infections are known to be less efficient than infections with other retroviruses, and transmission of free HTLV-1 is considered not to occur *in vivo*. However, it has been demonstrated that cell-free HTLV-1 virions can infect primary lymphocytes and dendritic cells *in vitro*, and that virions embedded in biofilms on cell membranes can contribute to transmission. The establishment of an efficient cell-free HTLV-1 infection model would be a useful tool for analyzing the replication process of HTLV-1 and the clonal expansion of infected cells. We first succeeded in obtaining supernatants with high-titer cell-free HTLV-1 using a highly efficient virus-producing cell line. The HTLV-1 virions retained the structural characteristics of retroviruses. Using this cell-free infection model, we confirmed that a variety of cell lines and primary cultured cells can be infected with HTLV-1 and demonstrated that the provirus was randomly integrated into all chromosomes in the target cells. The provirus-integrated cell lines were HTLV-1-productive. Furthermore, we demonstrated for the first time that cell-free HTLV-1 is infectious *in vivo* using a humanized mouse model. These results indicate that this cell-free infection model recapitulates the HTLV-1 life cycle, including entry, reverse transcription, integration into the host genome, viral replication, and secondary infection. The new cell-free HTLV-1 infection model is promising as a practical resource for studying HTLV-1 infection.

**IMPORTANCE:**

Co-culture of infected and target cells is frequently used for studying HTLV-1 infection. Although this method efficiently infects HTLV-1, the cell mixture is complex, and it is extremely difficult to distinguish donor infected cells from target cells. In contrast, cell-free HTLV-1 infection models allow for more strict experimental conditions. In this study, we established a novel and efficient cell-free HTLV-1 infection model. Using this model, we successfully evaluated the infectivity titers of cell-free HTLV-1 as proviral loads (copies per 100 cells) in various cell lines, primary cultured cells, and a humanized mouse model. Interestingly, the HTLV-1-associated viral biofilms played an important role in enhancing the infectivity of the cell-free infection model. This cell-free HTLV-1 infection model reproduces the replication cycle of HTLV-1 and provides a simple, powerful, and alternative tool for researching HTLV-1 infection.

## INTRODUCTION

Human T-cell leukemia virus type 1 (HTLV-1) is a human retrovirus that is the etiologic agent of adult T-cell leukemia (ATL) and HTLV-1-associated myelopathy/tropical spastic paraparesis (HAM/TSP) (1–5). The major endemic areas for HTLV-1 are South Japan, South America, Australia and Melanesia, Central Africa, and the Caribbean, and an estimated 10–20 million people worldwide are infected with this virus (6–9). HTLV-1 is transmitted vertically from infected mother to child primarily through breastfeeding (10), but transmission can also occur through *in utero* transmission (11), sexual contact (12), or blood transfusion.

HTLV-1 characteristically establishes a persistent infection in CD4-positive T cells *in vitro* and *in vivo* (13–16). Furthermore, co-cultures of cord blood lymphocytes and peripheral blood mononuclear cells (PBMCs) with HTLV-1-producing cells have yielded HTLV-1-infected B cells and CD8-positive T cells (17–20). HTLV-1 has been reported to infect many cell types *in vitro*, including monocytes (21), fibroblasts (22), endothelial cells (23, 24), microglial cells (21), and neurons (25). HTLV-1 entry into target cells requires the initial binding of the envelope protein gp46 to heparin sulfate proteoglycans and neuropilin 1 (NRP1) (26–28) and subsequent binding to glucose transporter 1 (GLUT1) (29). These molecules are ubiquitously expressed in a variety of cells, which may explain the wide range of target cells for HTLV-1.

Given that few infectious viral particles are detected in the peripheral blood of HTLV-1 carriers, including ATL and HAM/TSP patients, HTLV-1 infection is thought to result primarily from direct contact between infected lymphocytes and target cells, the formation of virological synapses (30), and the transfer of membrane-bound viruses embedded in biofilm-like structures on the surface of infected cells (31). Indeed, the infectivity of HTLV-1 produced by infected cells *in vitro* has been shown to be very low (32), and most studies of HTLV-1 infection and T cell transformation have used co-culture and syncytial formation assays (33). However, these experimental systems are limited in their ability to quantitatively analyze early infections and replication events in that (ⅰ) it is difficult to distinguish between the newly synthesized HTLV-1 products released by target infected cells and the viral products already present in donor infected cells; (ⅱ) the cell mixture is complex, and the complete removal of donor infected cells is difficult; and (ⅲ) the structure and nucleic acid sequence of the provirus in donor infected cells are not clear.

As an alternative, methods for cell-free HTLV-1 infection experiments have been developed. Cell-free HTLV-1 infection is known to be less efficient than infections of other retroviruses, and this has hindered the analysis of the HTLV-1 replication step. However, the infection of primary lymphocytes was first demonstrated with a cell-free infection system (14, 32), and cell-free HTLV-1 has been demonstrated to efficiently infect dendritic cells (34). Furthermore, virions produced by HTLV-1-infected T cells have been shown to pass from the apical membrane to the basal surface of epithelial cells within endosomes (35), and epithelial cells themselves can be infected with HTLV-1 and release virions from their basal surface (36, 37). More recently, it has been reported that virions embedded in biofilms can contribute to HTLV-1 transmission (38). These facts suggest that cell-free HTLV-1 not only plays a role in the spread and maintenance of persistent infection but can also be useful as an experimental source of infection. However, few studies have quantitatively evaluated the infectious titers of cell-free HTLV-1, and this idea remains controversial.

In this study, we aimed to establish a new cell-free HTLV-1 infection model. We succeeded in obtaining high titers of cell-free HTLV-1 by transducing an infectious HTLV-1 molecular clone into an optimized virus-producing cell line. This cell-free HTLV-1 infection model retained the structural characteristic of retroviruses and resulted in appreciable proviral loads against a variety of cell line types and primary cultured cells *in vitro*. We demonstrated that the proviruses were randomly integrated into all chromosomes in target cells *in vitro*. Furthermore, we demonstrated for the first time that cell-free HTLV-1 is infectious and can act as an *in vivo* source of infection by establishing a persistent infection in a humanized mouse model. The new cell-free HTLV-1 infection model may be useful to those investigating the biological and biochemical interactions between HTLV-1 and host cells.

## RESULTS

### Production of cell-free HTLV-1 virions and evaluation of their infectivity

**Fig 1:**
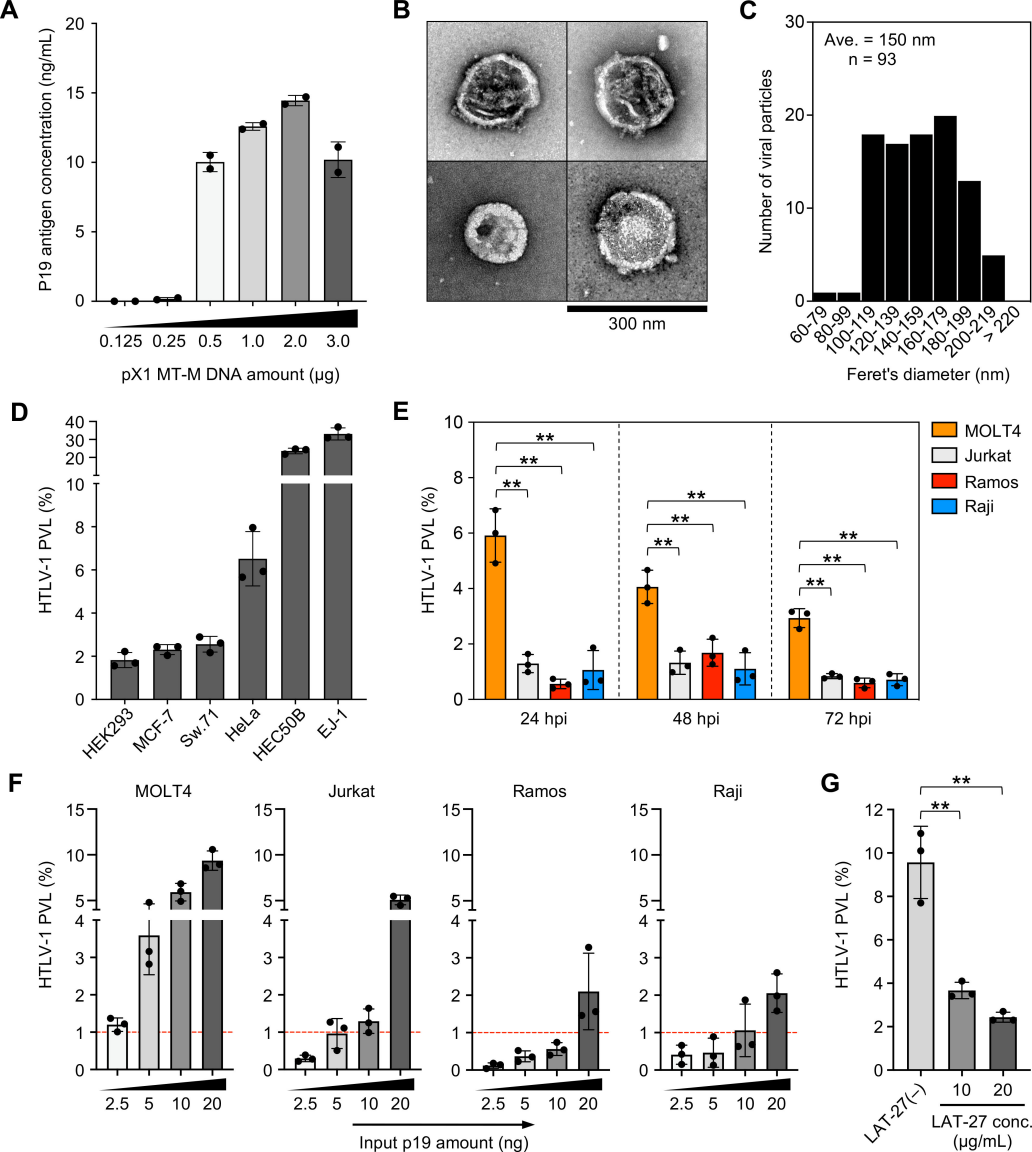
Establishment of HTLV-1 cell-free infection model (**A**) Determination of the optimal amount of pX1 MT-M, an infectious molecular clone of HTLV-1, for *in vitro* viral production. Approximately 0.5 × 10^6^ Lenti-X 293T cells were transfected with 0.125–3.0 µg of pX1 MT-M for 48 hours. HTLV-1-containing culture supernatants of transfected cells were harvested and quantified by HTLV-1 p19 ELISA. (**B**) Morphological evaluation of the obtained virions by transmission electron microscopy (TEM). HTLV-1 particles were negatively stained with 2% uranyl acetate. Scale bar = 300 nm. Similar images of HTLV-1 particles were obtained in two independent experiments. (**C**) Particle size distribution shown by the maximum diameters of spherical or oval virions obtained in TEM analysis. Diameters of 93 particles were measured by the ImageJ software, and the number of particles in each 20 nm range is shown. (**D and E**) Proviral load (PVL) of infected adherent and suspended cells using the cell-free infection model. The PVLs were analyzed at 48 hours post-infection (hpi) for the adherent cells shown in panel D and at 24, 48, and 72 hpi for the suspended cells shown in panel E. (**F**) The PVL of four suspended cell lines depending on the amount of virion. Virus levels were quantified by HTLV-1 p19 ELISA. The PVLs were analyzed at 48 hpi. (**G**) HTLV-1 Env-dependent infection was assessed by neutralizing assay. MOLT4 cells were infected using the cell-free infection model in the presence or absence of HTLV-1 envelope-specific neutralizing antibody (LAT-27, 10 or 20 µg/mL). The PVLs were analyzed at 48 hpi. In panels E and G, asterisks represent significant differences versus the data for MOLT4 and LAT-27(–), respectively (**P < 0.01 by one-way analysis of variance (ANOVA) followed by Dunnett's multiple comparison test). In panels D–G, the data are the means ± SD of three independent experiments.

In this study, we tried to develop a novel cell-free HTLV-1 infection system for cultured cells *in vitro*. To determine whether HTLV-1 viral production can be induced in culture conditions, we transfected Lenti-X 293T cells with the HTLV-1 proviral plasmid pX1 MT-M and cultured them for 48 hours. We then evaluated the amount of HTLV-1 p19 antigen in the culture supernatant, which reflects the amount of HTLV-1 virus produced. The concentration of HTLV-1 p19 antigen produced by the infectious molecular clone increased in a dose-dependent manner, ranging from 0.125 to 2.0 µg of pX1 MT-M, and peaked at 2.0 µg of the expression plasmid (Fig. 1A). To obtain more information on the cell-free HTLV-1 virion structure, we used transmission electron microscopy (TEM) with negative staining (39). Homogeneous virions that were spherical and oval shaped and ~100–200 nm in diameter were observed in the supernatant of transfected Lenti-X 293T cells (Fig. 1B and C). The mean particle size of 93 virions was 150 nm (Fig. 1C), which is in accordance with previous reports (40).

Next, we hypothesized that the addition of HTLV-1-containing culture supernatant might induce the *de novo* infection of target cultured cells. Six adherent cell lines were exposed to the culture supernatant of transfected cells, and the HTLV-1 proviral load was evaluated 48 hours later. HEC50B and EJ-1 cells showed a high proviral load, while HEK293, MCF-7, Sw.71, and HeLa cells showed a relatively low load (Fig. 1D), suggesting that differences in the proviral load reflected the susceptibility of each cell line to HTLV-1 infection.

As expected from the results for the adherent cell lines (Fig. 1D), proviral load was also detected in the four suspended cell lines after 24, 48, and 72 hours of exposure to the culture supernatant (Fig. 1E). The proviral loads of Jurkat, Ramos, and Raji cells were comparable at each time point, while those of MOLT4 cells were significantly higher at each time point compared with those of the other cells (Fig. 1E). In these four cell lines, the proviral load increased in a dose-dependent manner (Fig. 1F). The amount of virus required to reach a proviral load of 1% suggested that the susceptibilities of the four cell lines decreased from MOLT4, Jurkat, Raji, to Ramos (Fig. 1F). Furthermore, we performed infection inhibition experiments using an HTLV-1 envelope glycoprotein-specific neutralizing monoclonal antibody. A well-validated neutralizing antibody, LAT-27, was added to the culture supernatant (p19 = 10 ng equivalent of virus) and incubated at 37°C for 1 hour. Untreated culture supernatant was used as a control. HTLV-1 infection in MOLT4 cells was assessed by the proviral load at 48 hours post-exposure. The proviral load was significantly suppressed by LAT-27 treatment compared with the control (Fig. 1G), and this reduction in the proviral load was antibody concentration-dependent (61.6%–74.5% reduction). Infection was suppressed by neutralizing antibody, suggesting that cell-free HTLV-1 in the culture supernatant infects cells via an envelope-dependent manner.

These results suggest that the culture supernatants derived from Lenti-X 293T cells contained infectious cell-free HTLV-1 virions, which induced *de novo* infections in target cells cultured *in vitro*.

### Evaluation of HTLV-1 viral biofilms in cell-free infection model

**Fig 2:**
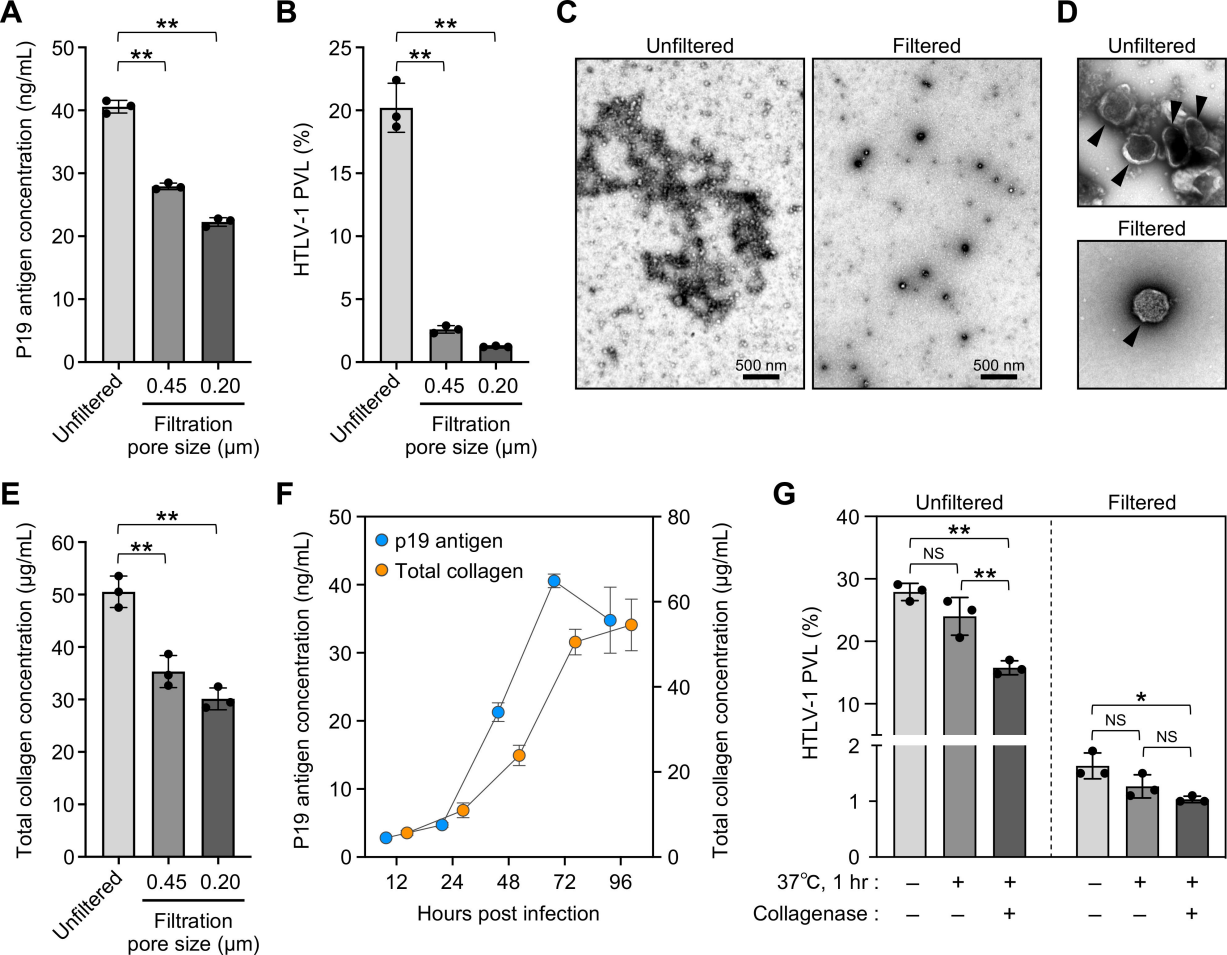
HTLV-1-associated viral biofilms contribute to the infectivity of cell-free infection model (**A and B**) Effect of filtration on virus levels and infectivity in the culture supernatant. After filtration with two pore sizes (0.45 and 0.20 µm), virus levels were quantified by HTLV-1 p19 ELISA and shown in panel A. MOLT4 cells were infected with unfiltered or filtered culture supernatants with the equivalent of 20 ng of HTLV-1 p19. The PVLs were analyzed at 48 hpi and shown in panel B. (**C and D**) Morphological evaluation of the virus assemblies by TEM. Unfiltered culture supernatants and filtered culture supernatants with a pore size of 0.20 µm were negatively stained with 2% uranyl acetate. Representative lower and higher magnification images of two independent experiments are shown in panels C and D, respectively. Black arrowheads show mature virus particles with electron-dense cores in panel D. Scale bars in panels C and one side of the square in panel D represent 500 nm. (**E**) Effect of filtration on the total collagen levels. After filtration with two pore sizes (0.45 and 0.20 µm), the amount of total collagen in the culture supernatant was quantified. (**F**) Kinetics analysis of the HTLV-1 p19 antigen levels and the amount of total collagen in culture supernatants. Approximately 3.5 × 10^6^ Lenti-X 293T cells were transfected with 20 µg of pX1 MT-M. Culture supernatants were harvested every 24 hours until 96 hours post-transfection. The HTLV-1 p19 antigen levels and the amount of total collagen were quantified and data are shown on the left and right y-axes, respectively. (**G**) Effect of collagenase treatment on infectivity in the culture supernatant. Unfiltered culture supernatants and filtered culture supernatants with a pore size of 0.20 µm were incubated at 37°C for 1 hour with or without recombinant collagenase (100 U/mL). After incubation, MOLT4 cells were infected with these culture supernatants with the equivalent of 20 ng of HTLV-1 p19. The PVLs were analyzed at 48 hpi. Asterisks in panels A, B, and E represent significant differences versus the data for unfiltered culture supernatant (**P < 0.01 by one-way ANOVA followed by Dunnett’s multiple comparison test). In panel G, significant differences were calculated with one-way ANOVA followed by Tukey’s multiple comparison test (*P < 0.05; **P < 0.01; and NS, not significant). In panels A, B, E, F, and G, the data are the means ± SD of three independent experiments.

HTLV-1 virions accumulated in the extracellular matrix structures have been reported to contribute to both the cell-to-cell and cell-free infection titers (31, 38). Since the size of such biofilm-like structures is usually much larger than that of virions (31), we hypothesized that strict filtration would affect the amount of biofilm in the culture supernatant. We therefore first examined whether filtration affects viral antigen levels and the infection titer. After filtration of culture supernatants through filters with two different pore sizes, the HTLV-1 p19 antigen levels in the supernatant were quantified and found to be significantly reduced (Fig. 2A). Infectivity was then examined by inoculating MOLT4 cells with the same amount of virus (p19 = 20 ng equivalent) after a similar filtration process. Interestingly, MOLT4 cells inoculated with the filtered virus showed a marked reduction in the proviral load (87.1%–93.9% reduction; Fig. 2B).

Next, we examined whether filtration altered the morphology of virus particles in the culture supernatant by TEM. In unfiltrated culture supernatants, we observed a mesh of electron-dense material among the viral particles, which may have been extracellular matrix (Fig. 2C). Furthermore, the size and shape of the biofilm-like structures were consistent with a previous report (31), and mature viral particle assemblies with electron-dense cores were observed within these structures (Fig. 2D), which were absent from filtered culture supernatants (Fig. 2C and D).

The extracellular biofilm-like structures associated with HTLV-1 are enriched in viral proteins (also called viral biofilms) and enriched in collagen and galectin-3 (31). In addition, collagen is overproduced in HTLV-1-infected cells via a Tax-mediated manner (41). We investigated the relationship between viral biofilm and total collagen levels in culture supernatants. As shown in Fig. 2E, filtration significantly reduced the amount of total collagen in the culture supernatant, coinciding with a decrease in the amount of HTLV-1 p19 antigen (Fig. 2A). We then transfected Lenti-X 293T cells with pX1 MT-M and monitored the amount of p19 antigen and total collagen in the culture supernatant over time. Both concentrations increased in a time-dependent manner, indicating a correlation between these factors (Fig. 2F).

Finally, we examined whether infectivity was affected by the degradation of collagen in the culture supernatant. To partially degrade the viral biofilm, recombinant collagenase was added to the culture supernatant, which was incubated at 37°C for 1 hour. The method for assessing infectivity was the same as described above. When infected with collagenase-treated unfiltered culture supernatants, the proviral loads were significantly reduced compared with the corresponding culture supernatant without collagenase, whereas the proviral loads were not altered in the filtered culture supernatants (Fig. 2G). There was no significant change in infectivity after 1 hour of incubation at 37°C with or without filtration. The degradation of collagen, which constitutes the HTLV-1-associated viral biofilm, was suggested to reduce the protection and adhesion of the viral particles, resulting in a partial reduction in infectivity in unfiltered culture supernatants. These results indicate that the viral biofilm contributes to the maintenance of infectivity in this cell-free infection model.

### Evaluation of HTLV-1 viral replication cycle in cell-free infection model

To examine whether the HTLV-1 viral replication cycle is reproduced in cells infected with this cell-free infection model, we analyzed the levels of HTLV-1 proviral integration, viral mRNA transcription, and viral protein translation in cells after exposure to culture supernatant containing HTLV-1.

**Fig 3:**
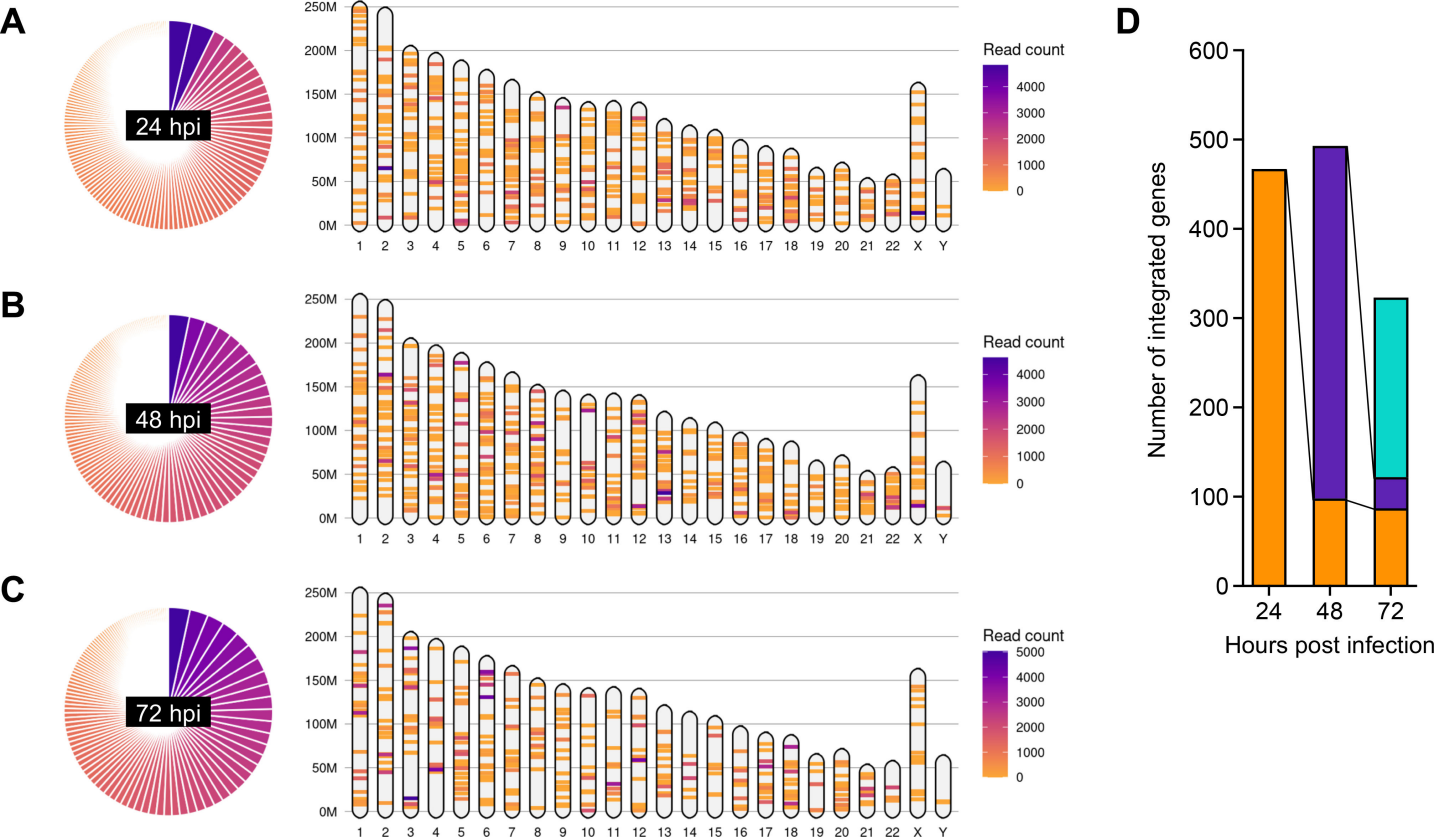
Integration of HTLV-1 proviral DNA in the cell-free infection model (**A–C**) HTLV-1 random integration into host genome was analyzed by RAISING. Read count proportions are shown in a pie chart (left), and the visualization of chromosome mapping of HTLV-1 integration sites is presented (right). Representative results obtained at 24, 48, and 72 hpi are shown in panels A, B, and C, respectively. (**D**) Kinetic analysis of HTLV-1-integrated genes in host genome. The number of genes detected for the first time at 24 hpi (orange), 48 hpi (purple), and 72 hpi (cyan) are presented.

As shown in Fig. 1F, MOLT4 cells were used to assess random proviral integration because of their high susceptibility to HTLV-1. HTLV-1 integration sites in the host genome were analyzed at 24-, 48-, and 72 hours post-infection (hpi), and the numbers of detected integration sites were 721, 747, and 618, respectively. Visualization of chromosome mapping of HTLV-1-integration sites and the clone size proportions revealed that HTLV-1 was randomly integrated into all chromosomes to form a large number of infected clones in the cell-free infection model (Fig. 3A through C). These data represent all integration sites in a polyclonal infected cell population. At 24 hpi, HTLV-1 was found to be inserted within 467 genes in the host genome. Of these 467 genes, 98 and 87 genes were commonly detected at 48 and 72 hpi, respectively (Fig. 3D). In addition, 395 new gene inserts were detected at 48 hpi, 35 of which were still common, and 201 new gene inserts were detected at 72 hpi (Fig. 3D). Furthermore, we analyzed the full-length proviral DNA sequences in serial samples taken at 24, 48, and 72 hpi (the same samples used in Fig. 3A through C). The HTLV-1 proviral sequence of infected MOLT4 cells was identical to the original sequence of the pX1 MT-M plasmid regardless of infection time. No clear nucleotide sequence variations or deletions were observed, including in the LTRs or in the coding regions of the *pX*, *Env*, and *HBZ* genes (data not shown). These results suggest that *de novo* HTLV-1 infections are repeated under culture conditions.

**Fig 4:**
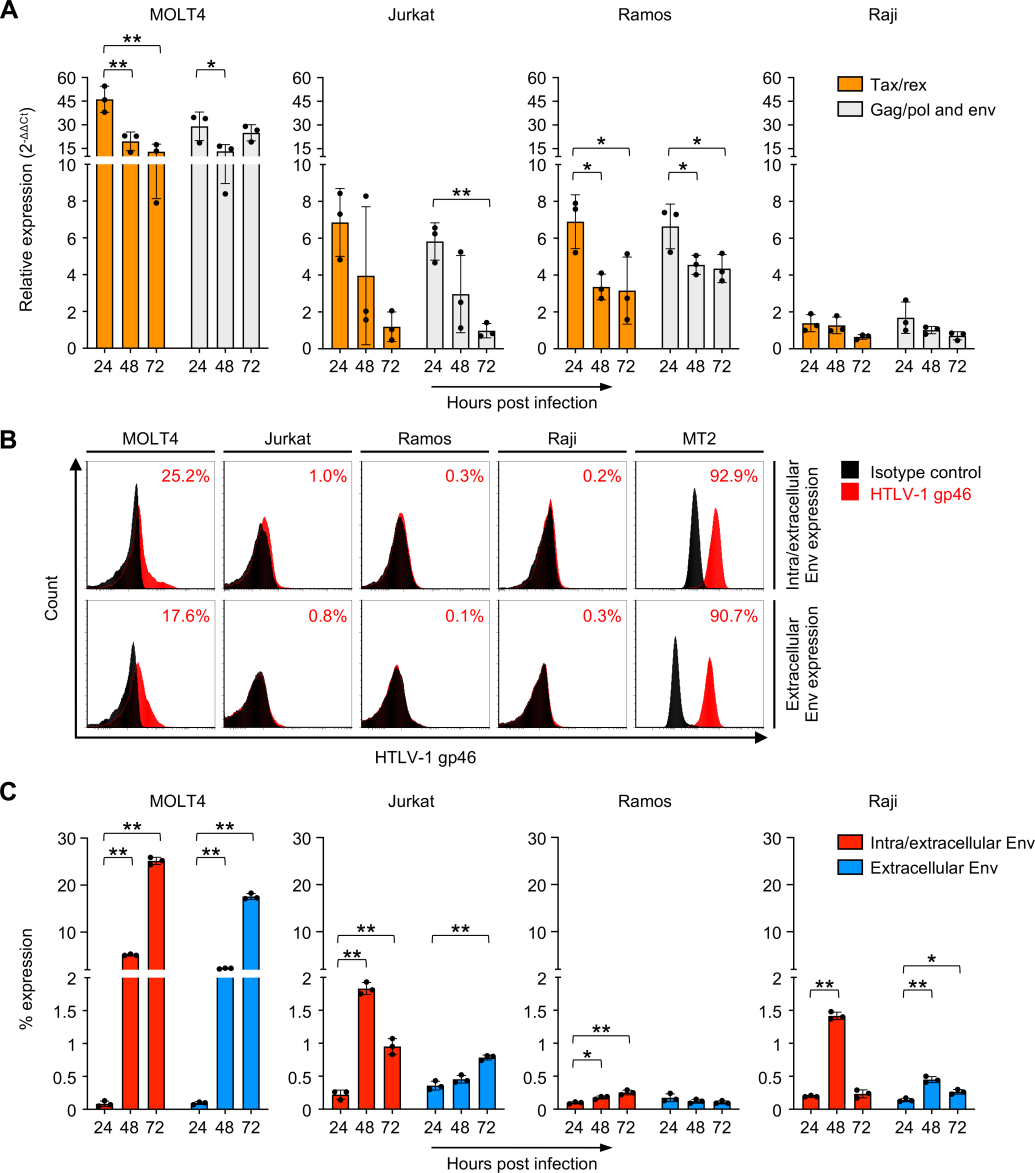
Evaluation of HTLV-1 viral transcription and translation in the cell-free infection model (**A**) Relative expression of *tax/rex*, and *gag/pol* and *env* mRNAs was evaluated by real-time PCR at 24, 48, and 72 hpi. The normalized expression levels for Raji cells at 72 hpi were set as one as a reference. The kinetic data are shown as the means ± SD of three independent experiments. (**B and C**) Both intra- and extracellular or only extracellular expression levels of HTLV-1 envelope glycoprotein were analyzed in suspended cell lines infected with cell-free infection model at 24, 48, and 72 hpi. MT2 cells were used as a positive control. The cells were stained with anti-HTLV-1 gp46 mAb (or mouse IgG1 mAb as an isotype control). Representative results and the percentages of HTLV-1 gp46-positive cells (right) obtained at 72 hpi are shown in panel B. The kinetic data are shown as the means ± SD of three independent experiments in panel C. Asterisks represent significant differences versus the data for 24 hpi for each cell line in panels A and C (*P < 0.05; **P < 0.01 by one-way ANOVA followed by Dunnett’s multiple-comparisons test).

We then analyzed viral mRNA expression and viral protein translation levels over time in four suspended cell lines, MOLT4, Jurkat, Ramos, and Raji cells. These cell lines were infected with HTLV-1 in the cell-free infection model. Multiple viral mRNAs (*tax/rex* mRNA, and *gag/pol* and *env* mRNA) and intracellular and extracellular envelope proteins were detected in all four cell lines (Fig. 4A and C). As shown in Fig. 4B, flow cytometric analysis of both intra- and extracellular or only extracellular Env proteins at 72 hpi revealed that HTLV-1-infected MOLT4 cells were 25.2% and 17.6% positive for intra/extracellular and extracellular envelope proteins, respectively. Consistent with their high susceptibility to HTLV-1, MOLT4 cells showed the highest viral mRNA expression and viral protein production, while Jurkat and Ramos cells showed more moderate levels (Fig. 4A and C). A time-dependent decrease in expression was observed in these three cell lines (Fig. 4A), while a gradual increase in the positivity rate of intra/extracellular and extracellular envelope proteins was observed (Fig. 4C). The expression of viral mRNA did not necessarily coincide with the production of viral proteins, suggesting a time lag between the two.

Taken together, these results indicate that the replication cycle of HTLV-1, including viral integration, transcription, and translation, is reproduced in infected cells in the cell-free infection model.

### Evaluation of susceptibility to primary cultured cells using the cell-free infection model

**Fig 5:**
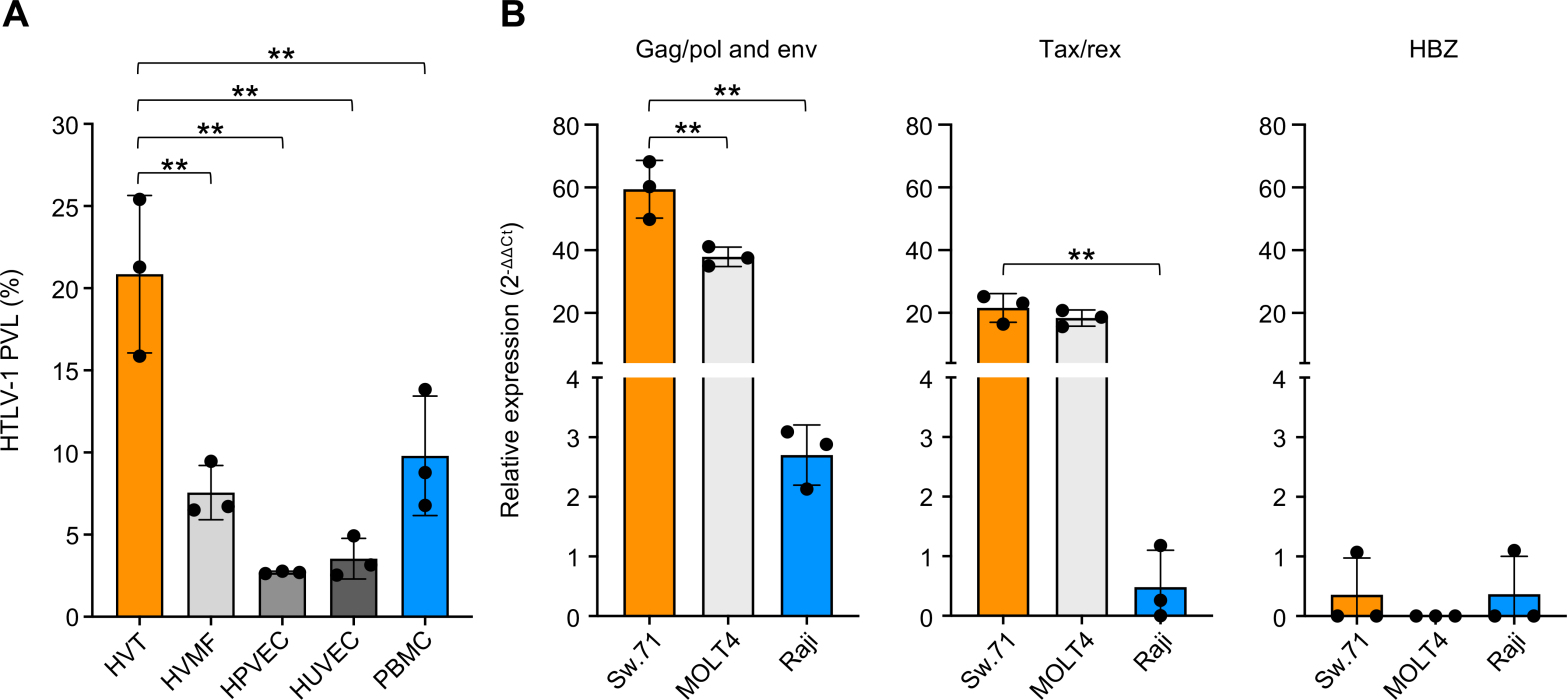
Assessment of HTLV-1 susceptibility of human primary cells in the cell-free infection model (**A**) Susceptibility of cells constituting the blood-placental barrier (HVT, HVMF, HPVEC, and HUVEC) and PBMCs to HTLV-1 in the cell-free infection model. The PVLs were analyzed at 48 hpi, and the data are the means ± SD of three independent experiments. (**B**) Relative expression of *gag/pol* and *env*, *tax/rex*, and *HBZ* mRNAs at 48 hpi were evaluated by real-time PCR in immortalized normal trophoblast (Sw.71), MOLT4, and Raji cells infected with HTLV-1 in the cell-free infection model. In panels A and B, asterisks represent significant differences versus the data for HVT and Sw.71, respectively (**P < 0.01 by one-way ANOVA followed by Dunnett’s multiple-comparisons test). HVT; human villous trophoblasts, HVMF; human villous mesenchymal fibroblasts, HPVEC; human placental vascular endothelial cells, HUVEC; human umbilical vein endothelial cells, PBMC; peripheral blood mononuclear cells.

Next, we examined whether the susceptibility of primary cultured cells to HTLV-1 infection could be evaluated using the cell-free infection model. We previously reported that, among the cells constituting the blood-placental barrier (human villous trophoblasts; HVT, human villous mesenchymal fibroblasts; HVMF, human placental vascular endothelial cells; HPVEC, and human umbilical vein endothelial cells; HUVEC) in the placenta of pregnant HTLV-1-carrier women, HVT are the most susceptible to HTLV-1 (11). However, past studies have not used authentic cell-free HTLV-1. We then evaluated the HTLV-1 susceptibility of primary cultures containing placental cells and PBMCs using our cell-free infection model, and HVT showed a significantly higher proviral load than the other placental cells and PBMCs (Fig. 5A), consistent with previous reports (11).

Furthermore, we used this cell-free infection model to infect human normal trophoblasts (Sw.71) and lymphoid cells (MOLT4 and Raji) with HTLV-1 and compared the viral mRNA expression levels. As expected from a previous report, *gag/pol* and *env,* and *tax/rex* mRNA expression levels were significantly higher in Sw.71 trophoblasts than lymphoid cells, while no significant differences in *HBZ* expression levels were observed (Fig. 5B). These results indicate that this cell-free infection model is useful for assessing primary cultured cell susceptibility to HTLV-1 infection.

### Evaluation of *in vivo* infectivity in cell-free infection model

The findings of high infectivity of HTLV-1 in cells exposed to the culture supernatants *in vitro* prompted us to conduct further studies to assess *in vivo* infectivity with the cell-free infection model. We previously established a humanized mouse model of HTLV-1 infection to evaluate viral pathogenicity and infectivity (42, 43). We therefore examined whether cell-free HTLV-1 causes the *de novo* infection of target T cells *in vivo* using the humanized mouse model.

**Fig 6:**
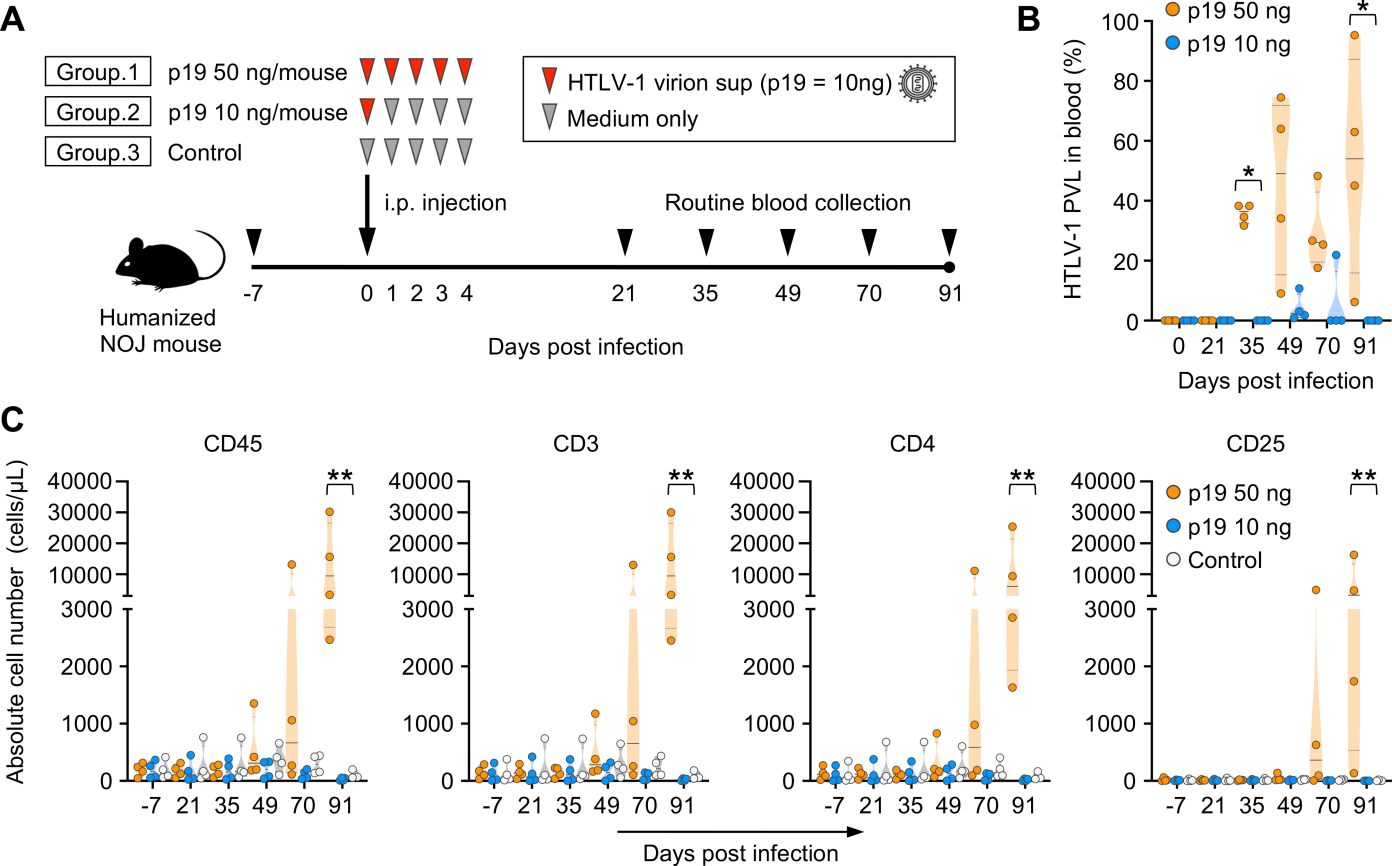
Cell-free HTLV-1 virions can induce *de novo* infection *in vivo* (**A**) Schematic of the experimental schedule. All NOJ mice were reconstituted with a human immune system by the intrahepatic transplantation of human CD133^+^ hematopoietic stem cells into newborn mice. After humanization, 12 mice were divided into three groups and inoculated intraperitoneally with HTLV-1-containing culture supernatants or Dulbecco’s Modified Eagle Medium as a control (p19 50 ng/mouse group, *n* = 4; p19 10 ng/mouse group, *n* = 4; control group, *n* = 4). Black arrowheads indicate the timepoints of blood collection. (**B**) Quantification of HTLV-1 PVL in the peripheral blood of inoculated mice. HTLV-1 PVL was determined by real-time PCR at 0-, 21-, 35-, 49-, 70-, and 91-day post-infection. One dot represents the result of an individual mouse. (**C**) Human CD45^+^ leucocytes, CD3^+^ lymphocytes, total CD4^+^ T cells, and CD25^+^ T cells were routinely analyzed by flow cytometry. One dot represents the result of an individual mouse. The absolute numbers of human CD45^+^, CD3^+^, CD4^+^, and CD25^+^ leucocytes are shown in panel C. CD3^+^ lymphocytes were gated to analyze the populations of CD4^+^ and CD25^+^ T cells. Asterisks in panel B represent significant differences between groups given p19 50 ng and p19 10 ng per mouse (*P < 0.05 by Mann–Whitney *U*-test). Asterisks represent significant differences among three groups in panel C (**P < 0.01 by Kruskal–Wallis test followed by Dunn’s multiple-comparisons test).

As shown in Fig. 6A, 12 humanized mice were divided into three groups of four mice per group (two males and two females), with two groups given doses of HTLV-1 p19 (50 or 10 ng per mouse) and a control group. Blood samples were routinely collected every 21 days after inoculation of the humanized mice with the culture supernatant. The proviral load was analyzed by real-time PCR, and cell population and cell count were monitored by flow cytometry.

On day 35 post-inoculation, the proviral load was significantly increased in mice in the 50 ng group compared with that in the 10 ng group (Fig. 6B). On day 91 post-inoculation, the proviral load reached almost 100% in some mice in the 50 ng group, whereas almost no proviral load was detected in mice in the 10 ng group (Fig. 6B). Furthermore, the numbers of blood human leukocytes and T cells, including CD4-positive and CD25-positive cells, were significantly increased in the 50 ng group mice (Fig. 6C). In contrast, no changes to human leukocyte counts or T-cell phenotype were observed over time in mice in the 10 ng or control groups (Fig. 6C). These results indicate that the HTLV-1 in the culture supernatants efficiently infected human lymphocytes, even *in vivo*, resulting in the abnormal proliferation of infected T cells.

## DISCUSSION

In previous studies, various attempts to establish cell-free HTLV-1 infection models have been reported. Sources of virus to date have been culture supernatants of HTLV-1-infected cell lines (14, 32, 38, 44), culture supernatants or concentrates of cells transduced with infectious molecular clones (45, 46), or preparations provided by manufacturers (47), and the results obtained with these have been inconsistent. Analyses of virions in the culture supernatants of MT2 and C91/PL cells, representative productive HTLV-1-infected cell lines, have provided important information on cell-free HTLV-1 infection titers and biochemical and morphological characteristics (48–50). The outer diameter of HTLV-1 particles is 80–160 nm, and HTLV-1 are characterized morphologically as mature C-type viruses similar to animal-derived leukemia viruses (40). In the cell-free HTLV-1 infection model used in this study, we observed mature HTLV-1 virions in the culture supernatants. The morphological characteristics of these particles were identical to those of the virus produced in MT2 cells (40, 48). Virus-like particles (VLPs) released by cells expressing only the HTLV-1 Gag protein were reported to be slightly smaller than these virions, with diameter in the range of 110–120 nm (51, 52). This discrepancy may be due to differences between VLPs and authentic viral particles. In addition, the identification of virions and exosomes in the culture supernatants requires further investigation.

HTLV-1-transformed cell lines are capable of producing infectious virions. However, these cell lines have a high proportion of defective proviruses (53), and the cell-free viruses released have low infectivity. In fact, the first quantitative study estimated that only one in a million virions produced by MT2 cells is infectious (32). Although HTLV-1-infected cell lines can be a stable source of the virus, it is difficult to modify the provirus internal sequence, and cell-free HTLV-infection systems utilizing infectious molecular clones have been established. In previous studies, HTLV-1 proviruses were cloned from several groups, and the infectivity of the constructed provirus clones was demonstrated (54–56). Full-length proviruses derived from transformed cell lines CS-1 and CR (18, 57), obtained by co-culturing HTLV-1-infected cells from ATL patients from the USA with healthy human cord blood lymphocytes, were cloned, and the infectious molecular clones pCS-HTLV and pACH were respectively constructed (54, 55). Similarly, the infectious molecular clone K30p was constructed from the transformed cell line RH/K30, which was established by co-culturing MT2 cells with rabbit PBMCs (56). These infectious molecular clones have been widely used to study HTLV-1 transformation and infectivity *in vitro*. Of the three infectious molecular clones reported, pACH and K30p showed infectivity when co-cultured with transfected and target cells (54, 56).

Notably, Derse et al. demonstrated that HTLV-1 virions were released from pCS-HTLV-transfected cells and that cell-free HTLV-1 infected and immortalized primary T cells *in vitro* (55). They further showed that the pCS-HTLV-based VSV-G pseudotype virus was ~1,000-fold less infectious than a similar HIV-1-based VSV-G pseudotype virus (58). However, the temperature stabilities of HTLV-1 and HIV-1 virions were comparable to those of other retroviruses, indicating that the lower infectivity of the HTLV-1 virions was not due to their unusual instability (58). Although the stability of the HTLV-1 virion and envelope protein is controversial (59), the fact that the HTLV-1 virion remained infectious after DNase pretreatment of the virus solution and inoculation into mice in our experiments supports the theory that the stability of the HTLV-1 virion is comparable to that of other retroviruses.

Missense or nonsense mutations are scattered in the proviral internal sequence of ATL patients (60). ATL-derived pCS-HTLV has a premature stop codon in the p12 ORF, resulting in a defect in p12 translation (61). This defect was repaired by constructing the infectious molecular clone pCS-X1MT, in which the p12 and p30 ORFs were replaced by homologous sequences expressed in MT2 cells (61). In addition, molecular clones from CS-1 cells were shown to have four amino acid mutations from the consensus sequence in the *pol* gene (62). Mitchell et al. investigated whether mutations in the *pol* gene sequence encoding reverse transcriptase correlated with viral infectivity and found that viral titers decreased in almost direct proportion to the number of amino acid mutations from the consensus sequence (62). Conversely, recombinant viruses derived from MT2 and others, whose *pol* genes were closest to the consensus sequence, showed the highest titers. The infectious molecular clone used in this study, pX1MT-M, in which the reverse transcriptase gene-coding region of pCS-X1MT was replaced with the MT2-derived consensus sequence, showed a 10-fold higher infection titer than the original infectious molecular clone, pCS-HTLV (62). This optimized infectious molecular clone may contribute to the cell-free infection model in this study.

Despite the productive release of infectious virions *in vitro*, virions are rarely detected in the body of HTLV-1 carriers (37), suggesting that free virions do not contribute to the spread of *in vivo* infections. However, the importance of cell-free HTLV-1 in the development of associated diseases remains unclear. Recently, it has been reported that HTLV-1 virions accumulate in biofilm-like structures formed on the plasma membrane of infected cells by the accumulation of highly glycosylated proteins (31). Viral biofilms not only physically protect HTLV-1 envelope proteins but may also participate in intercellular adhesion and assist in the efficient binding of HTLV-1 virions to their receptors (59, 63). Viral biofilms are aggregates of HTLV-1 virions embedded in an extracellular matrix and are therefore significantly more infectious than free virions (38). Such biofilms are released spontaneously into the culture supernatant of infected cells and can also detach from infected cells, which may contribute to the infectivity titer of cell-free HTLV-1. However, viral biofilms in culture supernatants are removed by filtration. In most cell-free HTLV-1 infection models reported in the past, viruses were purified by filtration (14, 32, 38, 46, 58, 62), which likely removed the viral biofilms. The new infection model reported here retains any viral biofilms because the culture supernatant is not filtered.

In addition, the Lenti-X 293T cell line was used for the packaging cells in this study. Although the components and amount of these cells' extracellular matrix remain to be investigated, this cell line is useful for cell-free HTLV-1 production because it shows higher viral production than cell lines frequently used to date (64). Tax proteins have been reported to enhance retro- or lentivirus production (65), but surprisingly, had no effect on the Lenti-X 293T cell line (data not shown). Taken together, the cell-free HTLV-1 infection model established in this study has three advantages: (ⅰ) an optimized infectious molecular clone, (ⅱ) a cell-free infection source that retains the viral biofilm, and (ⅲ) a packaging cell line suitable for retrovirus production.

Cell-free HTLV-1 infectivity titers have been examined by various methods, including firefly luciferase activity (38, 58), reporter protein fluorescence intensity (38, 58), and syncytial plaque counts (44, 59). However, the widely accepted clinical methods for measuring HTLV-1-infected cells are the proviral load (number of viral copies per cell) and clonality based on integration sites. In the present study, we successfully assessed the cell-free HTLV-1 infection titer as a percentage of the proviral load in a variety of cell lines and primary cell cultures. Among lymphoid cell lines, MOLT4 cells were the most HTLV-1-susceptible, which is in accordance with previous reports (32, 55). HTLV-1 proviruses were integrated into all chromosomes in MOLT4 cells infected with this cell-free HTLV-1 infection model. Furthermore, we successfully observed the number and type of provirus-integrated genes over time after infection. Newly provirus-integrated genes were detected every 24 hpi in MOLT4 cells, suggesting there were sequential *de novo* infections *in vitro*. Similarly, the higher expression of viral mRNA and protein in MOLT4 cells every 24 hpi compared with that in other cell lines also supports the theory that *de novo* infection and completion of the viral replication cycle occurred *in vitro*.

Interestingly, many infected clones appeared to be generated and lost over time after infection of MOLT4 cells. Tax is not only essential for viral replication but is also involved in multiple aspects of infected cell proliferation and apoptosis (66). Tax promotes cell proliferation and transformation, but in contrast, over-activation of NF-κB by Tax has been reported to cause rapid cellular senescence in most cells (67). We plan to analyze the single-cell expression of infected cells in the cell-free HTLV-1 infection model. Clonality analysis based on the proviral integration site is difficult in the early stages of infection in the co-culture model of HTLV-1 infection because the donor infected cells cannot be eliminated. Thus, our cell-free HTLV-1 infection model provides a useful tool for the analysis of viral replication and clonal dynamics of infected cells in the early stages of infection. In addition, this cell-free HTLV-1 infection model can be used to assess various primary cells' susceptibility to HTLV-1, contributing to the elucidation of transmission routes.

Importantly, we have demonstrated for the first time using a humanized mice model that cell-free HTLV-1 is infectious *in vivo*. Mice inoculated with 10 ng of cell-free virus were not infected, but mice inoculated with 50 ng of cell-free virus showed a persistent infection, indicating that dose was an important factor. Generally, to infect humanized mice with HTLV-1, infected cells are inoculated directly into the mice (68). We have previously shown that the peripheral blood proviral load was detected after 14 days in humanized mice were inoculated with MT2 cells (42). In contrast, in humanized mice inoculated with 50 ng of cell-free virus, the peripheral blood proviral load was undetectable after 21 days and only detected after 35 days. Human leukocytosis was also more severe in humanized mice inoculated with MT2 cells. Although dependent on the characteristics of the HSC donor, these facts suggest that the inoculation of persistently HTLV-1-infected cells such as MT2 may cause a more rapid spread of infection *in vivo*. However, there are many factors to consider in the direct inoculation of infected cells, such as the amount of virus produced in HTLV-1-infected cells, viral titer, and the persistence of infected cell lines, which leaves the issues of reproducibility and reliability. Furthermore, it is difficult to generate recombinants based on the exact sequence of a HTLV-1 provirus in chronically infected cell lines such as MT2. The cell-free HTLV-1 infection model established in this study enables strict characterization of the virus and is expected to provide superior operability and reproducibility in *in vivo* infection experiments.

HTLV-1 virions have traditionally been considered to have low infectivity. In fact, the infectivity of HTLV-1 virions derived from pX1-MT-M has been estimated to be 100-fold less than that of HIV-1 virions (62). However, as mentioned above, it is likely that HTLV-1 virions maintain their infectivity by accumulating protective viral biofilms. Our cell-free HTLV-1 infection model has demonstrated infectivity *in vitro* and *in vivo* and could be used as a practical method of HTLV-1 infection in the future.

## MATERIALS AND METHODS

### Cell lines

Human adherent cell lines, HEK293, MCF-7, Hela, HEC50B, and EJ-1 cells, were purchased from the Japanese Collection of Research Bioresources (Osaka, Japan), and Lenti-X 293T cells were purchased from Takara Bio USA, Inc. (Mountain View, CA). These cells were cultured in Dulbecco's Modified Eagle's Medium (DMEM) (Sigma-Aldrich, St. Louis, MO) supplemented with 10% fetal bovine serum (FBS) and 1× antibiotic-antimycotic (Thermo Fisher Scientific, Waltham, MA). The human non-adherent cell lines Jurkat, MOLT4, Raji, and Ramos cells were cultured in RPMI-1640 medium (Sigma-Aldrich) supplemented with 10% FBS and 1× antibiotic-antimycotic. Until the cultures were 70%–80% confluent, the cells were passaged to a dilution of one-tenth to one-fifth. All cells were cultured under an atmosphere containing 5% CO_2_ at 37°C.

### Primary human cells

Human primary placental cells, including human villous trophoblasts (HVT), human villous mesenchymal fibroblasts (HVMF), and human placental vascular endothelial cells (HPVEC), and human umbilical vein endothelial cells (HUVEC) were purchased from Sciencell Research Laboratories (SCR, Carlsbad, CA) and cultured under the manufacturer's recommended conditions. Immortalized normal villous trophoblast cells, Sw.71 (69), were purchased from Applied Biological Materials (APB, Richmond, Canada) and cultured under the manufacturer's recommended conditions. Cells from one lot were used for downstream experiments. The following optimized culture media for each cell type were used: Trophoblast Medium (SCR) for HVT, Fibroblast Medium (SCR) for HVMF, and Endothelial Cell Medium (SCR) for HPVEC and HUVEC, and Prigrow IV Medium (APB) for Sw.71. All cells were cultured under an atmosphere containing 5% CO_2_ at 37°C.

### Cell-free infection model

A plasmid containing the HTLV-1 infectious molecular clone, pX-1 MT-M, was transfected into Lenti-X 293T cells using TransIT-LT1 Reagent (Mirus, Madison, WI) according to the manufacturer's recommended conditions. Lenti-X 293T cells were seeded at 3.5 × 10^6^ cells into a 10 cm culture dish 1 day before transfection, and the next day, the transfection mixture (TransIT-LT1; 60 µL, pX-1 MT-M; 20 µg, OptiMEM; 1,000 µL per 10 cm dish) was added. Six- and 24 hours post-transfection, the cells were washed gently with PBS (10 mL) to remove transfection mixture. At 48 hours post-transfection, all culture supernatants containing HTLV-1 virions were collected. To remove residual cell debris, the culture supernatant was centrifuged at 1,500 × *g* for 10 minutes and collected. To remove residual plasmid, DNase I (Thermo Fisher Scientific) was added to the culture supernatant (final concentration; 200 U/mL) and incubated for 1 hour at 37°C. The HTLV-1-containing culture supernatant was stored at −80°C until used as a viral stock solution. To infect target cells with HTLV-1, all cells were plated in 6-well culture plates (1.5 × 10^5^ cells per well), and viral stock solution was added to the wells (HTLV-1 p19 antigen = 10 or 20 ng equivalent of virus). In addition, the viral stock solution was preincubated with or without the HTLV-1 envelope-specific neutralizing monoclonal antibody (70), LAT-27 (10 or 20 µg/mL) for 1 hour at 37°C. To deplete extracellular matrix structures, the viral stock solution was filtrated through a 0.45 or 0.20 µm filter (Sartorius, Göttingen, Germany) before infection. To degrade viral biofilms, recombinant collagenase (Fujifilm Wako, Osaka, Japan) was added to the viral stock solution (final concentration; 100 U/mL) and preincubated for 1 hour at 37°C. These treated viral solutions were also used for HTLV-1 infection. At 48 hpi, the cells were harvested and collected in 15 mL tubes containing 10 mL DMEM and gently suspended. The cell mixture was centrifuged at 1,500 × *g* for 10 minutes, and the supernatant was removed. These procedures were repeated twice in total to wash the cells completely. After the wash step, the cell pellet was placed in a 1.5 mL tube with 1 mL DMEM, centrifuged at 1,500 × *g* for 10 minutes, and used for downstream experiments.

### Establishment of humanized mice and HTLV-1 infection

NOD/SCID Jak3-knockout (NOJ) mice were purchased from the KYUDO Company (Saga, Japan). These mice were maintained under specific pathogen-free conditions and handled in accordance with the institutional guidelines for animal experimentation at the National Institute of Infectious Diseases. CD133^+^ hematopoietic stem cells were purified from human cord blood (provided by the Japanese Red Cross Kanto-Koshinetsu Cord Blood Bank, Tokyo, Japan) with the CD133 MicroBead Kit for Hematopoietic Tissue (Miltenyi Biotec, Bergisch Gladbach, Germany) according to the manufacturer's instructions. Cells with a purity greater than 95% were immediately transplanted intrahepatically into newborn NOJ mice aged 0–2 days (0.5–1.0 × 10^5^ cells per mouse) or stored at −80°C until used as previously described (43). Twenty weeks after transplantation, 12 humanized mice were divided into three groups, an HTLV-1 p19 50 ng group, HTLV-1 p19 10 ng group, and control group. The numbers of female and male mice in each group were the same. Each experimental group was inoculated with culture supernatant with HTLV-1 virions by transperitoneal injection to establish HTLV-1 infection. The control group was inoculated with culture supernatant without HTLV-1 virions. The culture supernatant was administered for five consecutive days, with a maximum dose of HTLV-1 p19 10 ng per injection.

### HTLV-1 p19 antigen ELISA

The amount of HTLV-1 p19 antigen in culture supernatant was quantified using the HTLV-1 p19 Antigen ELISA Kit (ZeptoMetrix, Buffalo, NY) according to the manufacturer's instructions with the following three modifications. First, wells were filled with 200 µL PBS and pre-incubated at room temperature for 30 minutes before sample application. Second, samples were pre-diluted 30 times with PBS. Third, the substrate incubation time was 15 minutes at room temperature. After the procedure, absorbance at 450 nm was measured with the SpectraMax 190 Microplate Reader (Molecular Devices, San Jose, CA).

### Quantification of total collagen content

The amount of total collagen in culture supernatant was quantified using the Total Collagen Assay Kit (QuickZyme Biosciences, Leiden, Netherlands) according to the manufacturer's instructions. Absorbance at 570 nm was measured with the SpectraMax 190 Microplate Reader (Molecular Devices, San Jose, CA).

### Transmission electron microscopy

Culture supernatant containing HTLV-1 virions was filtrated through a 0.45 µm filter and purified using the Lenti-X Maxi Purification Kit (Takara Bio USA) according to the manufacturer's instructions. The original culture supernatant or purified virions resuspended in buffer were dispersed onto a 400-mesh, carbon film-supported Cu grid (NEM, Tokyo, Japan) for 10 seconds, fixed with 2% glutaraldehyde, washed with PBS, and negatively stained with 2% uranyl acetate solution for 10 seconds. TEM was performed at the Hanaichi UltraStructure Research Institute (Okazaki, Aichi, Japan) as described previously (39). Images of the virions were obtained using a TEM (H-7600; Hitachi, Tokyo, Japan). TEM image magnification ranged from 10,000 to 50,000 times. Fiji (ImageJ) software was used to measure the Feret's diameter of the particles. Diameter distribution and histogram analysis were performed with GraphPad Prism 8 (GraphPad Software, La Jolla, CA).

### Quantitation of HTLV-1 proviral load

To quantitate HTLV-1 infection, the copy number of proviral DNA was measured using a real-time PCR method, as previously reported (71). Genomic DNA was extracted from target cells using a QIAamp DNA Mini Kit (Qiagen, Hilden, Germany) according to the manufacturer's instructions. DNA samples were stored at −80°C until measurement of HTLV-1 proviral DNA levels. Quantitative real-time PCR was performed in duplicate to measure the copy numbers of the *pol* region of the HTLV-1 provirus and of the human RNase P (*RPPH1*) gene as an internal control. The primers and probe for HTLV-1 *pol* were as follows: 5′-GATCCCATCTCCAGGCTCAA-3′ (forward), 5′-TCCGCAATGGGTGAAACTG-3′ (reverse), and FAM-5′-CTCTCACAGATGCCCTA-3′-NFQ-MGB (probe). The mixture of primers and the probe for RPPH1 was purchased from Thermo Fisher Scientific (TaqMan Copy Number Reference Assay, human, RNase P). The proviral load was calculated as [(copy number of *pol*)/(copy number of *RPPH1*/2)] × 100 and expressed as *pol* copies per 100 cells (%).

### HTLV-1 random integration analysis

MOLT4 cells (9.0 × 10^5^ cells) were seeded into a 10 cm culture dish and infected with HTLV-1 viral stock solution (HTLV-1 p19 = 60 ng per dish). Cells were harvested every 24 hours until 72 hpi from the same plate. Genomic DNA was extracted from MOLT4 cells using a QIAamp DNA Mini Kit (Qiagen), and residual RNA in the extracted DNA was removed using RNase A (Thermo Fisher Scientific) according to the manufacturer's instructions. HTLV-1 random integration analysis by RAISING method and visualization of the chromosome mapping of HTLV-1 integrated genes were conducted at Fasmac Co., Ltd (Kanagawa, Japan) (72). HTLV-1 proviral sequence analysis was performed as described previously (73).

### Gene expression analysis

Total RNA was extracted from Sw.71, Jurkat, MOLT4, Ramos, and Raji cells using the RNeasy Mini Kit according to the manufacturer's instructions (Qiagen). To analyze gene expression levels, SYBR Green-based one-step RT-PCR was performed as described previously (74). Human glyceraldehyde-3-phosphate (*GAPDH*) was used as an internal control gene. The sequences of the forward and reverse primers were as follows: *Gag-pol* and *env*, 5′-TAGCAAACCGTCAAGCACAG-3′ and 5′-TGGACGGGCTATTATCCTTG-3′; *Tax/Rex*, 5′-ATCCCGTGGAGACTCCTCAA-3′ and 5′-AACACGTAGACTGGGTATCC-3′; HBZ, 5′-AGAACGCGACTCAACCGG-3′ and 5′-TGGCACAGGCAGGCATCG-3′; *GAPDH*, 5′-TTCTTTTGCGTCGCCAGCCGA-3′ and 5′-GTGACCAGGCGCCCAATACGA-3′. Data were collected using QuantStudio 5 Real-Time PCR (Thermo Fisher Scientific), and relative expression levels were calculated using the 2^–ΔΔCt^ method.

### Flow cytometry

To evaluate the intracellular and extracellular expression levels of the HTLV-1 envelope glycoprotein gp46, flow cytometric analysis was performed as described previously (43). Briefly, for intracellular staining of HTLV-1 envelope glycoprotein gp46, cell pellets were fixed in 4% paraformaldehyde for 20 minutes at room temperature. After washing with PBS twice, the cell pellets were permeabilized with 1% Tween for 5 minutes at room temperature. After again washing the cells with PBS twice, they were stained with anti-HTLV-1 gp46 mouse monoclonal antibody (mAb) (clone 67/5.5.13.1; Abcam, Cambridge, UK), mouse IgG1 isotype control mAb (clone 15-6E10A7; Abcam), and a phycoerythrin-conjugated anti-mouse IgG goat polyclonal antibody (Abcam), according to the manufacturer's recommendations.

To evaluate the populations of human lymphocytes in humanized mice, flow cytometric analysis was performed as previously reported (11). PBMCs were collected from mice, and then anti-human CD45-PE-Cy7 (clone HI30), CD3-FITC (clone OKT3), CD4-PE (clone OKT4), and CD25-APC (clone BC96) mouse mAbs (Cytek Biosciences, Fremont, CA) were used to stain cell surface markers according to the manufacturer's recommendations. All stained cells were immediately analyzed using the BD Accuri C6 flow cytometer with CFlow software (Becton Dickinson, Franklin Lakes, NJ), and the collected data were analyzed in FCS express 4 (*De Novo* Software, Los Angeles, CA).

### Statistical analysis

Differences between groups were assessed using the Mann–Whitney *U* test, the Kruskal–Wallis test followed by Dunn's multiple-comparisons test, and one-way analysis of variance followed by Dunnett's or Tukey's multiple-comparisons test. All statistical analyses were performed using GraphPad Prism 8 (GraphPad Software). *P* values less than 0.05 were considered statistically significant.

## Data Availability

The sequence of the pX1 MT-M plasmid is provided in the supplemental material.

## SUPPLEMENTAL MATERIAL

Supplemental material jvi.01862-23-s0001.fa Sequence of the pX1 MT-M plasmid.

ASM does not own the copyrights to Supplemental Material that may be linked to, or accessed through, an article. The authors have granted ASM a non-exclusive, world-wide license to publish the Supplemental Material files. Please contact the corresponding author directly for reuse.
